# Antibodies against multiple post-translationally modified proteins aid in diagnosis of autoimmune hepatitis and associate with complete biochemical response to treatment

**DOI:** 10.3389/fmed.2023.1195747

**Published:** 2023-07-25

**Authors:** Michelle D. van den Beukel, Anna E. C. Stoelinga, Adriaan J. van der Meer, Stef van der Meulen, Lu Zhang, Maarten E. Tushuizen, Bart van Hoek, Leendert A. Trouw

**Affiliations:** ^1^Department of Immunology, Leiden University Medical Center, Leiden, Netherlands; ^2^Department of Gastroenterology and Hepatology, Leiden University Medical Center, Leiden, Netherlands; ^3^Department of Gastroenterology and Hepatology, Erasmus Medical Center, Rotterdam, Netherlands

**Keywords:** autoantibodies, autoimmune hepatitis, autoimmune liver disease, post-translational modifications, epidemiology

## Abstract

**Background:**

(Auto)immune mediated and cholestatic liver disease (AILD) includes autoimmune hepatitis (AIH), primary biliary cholangitis (PBC) and primary sclerosing cholangitis (PSC). Especially AIH is characterized by the presence of autoantibodies and elevated serum immunoglobulins. In rheumatoid arthritis, autoantibodies against post-translational modifications (PTMs) such as citrullination (Cit) and carbamylation (CarP) are used as diagnostic and prognostic markers, respectively. We studied the presence of six anti-PTM antibodies in patients with the three AILDs and non-AILD.

**Methods:**

Antibodies against six PTMs (malondialdehyde–acetaldehyde adducts (MAA), advanced glycation end-products (AGE), CarP, acetylation (AL), Cit, and nitration (NT)) were tested in sera of patients with AILD (*n* = 106), non-AILD (*n* = 101) and compared with healthy controls (HC) (*n* = 100). Levels and positivity were correlated with clinical and biochemical features in a well-defined cohort of untreated AIH patients.

**Results:**

Anti-PTM antibodies were more often detectable in sera from AILD patients compared with HCs (anti-MAA: 67.9% vs. 2.0%, anti-AGE: 36.8% vs. 4.0%, anti-CarP: 47.2% vs. 5.0% and anti-AL: 18.9% vs. 5.0%). In untreated AIH, time to complete biochemical response (CBR) was associated with anti-MAA, anti-AGE, anti-CarP and anti-AL antibodies. Significantly more patients with at least three anti-PTM antibodies attained CBR at 12  months of treatment (13 vs. 3 *p* = 0.01).

**Conclusion:**

Anti-PTM antibodies are frequently present in AILD. The presence of anti-MAA, anti-AGE and anti-CarP antibodies correlates with the presence of AIH within this cohort. In AIH, harboring at least three anti-PTM antibody responses is positively associated with CBR. Determination of anti-PTM antibodies in liver disease may have diagnostic and prognostic value.

## Highligts

- Antibodies against post-translational modifications (anti-PTM antibodies) are used as diagnostic and prognostic markers in several autoimmune diseases, such as rheumatoid arthritis
- This study shows that these antibodies are often present in autoimmune mediated liver disease
- Compared to cholestatic liver disease, in autoimmune hepatitis most patients harbor antibodies against multiple post-translational modifications
- Such a multiple positivity was associated with complete biochemical response after 12 months of treatment in autoimmune hepatitis patients

## 1. Introduction

(Auto)immune mediated and cholestatic liver disease (AILD) is a heterogeneous group of both cholestatic and hepatocellular diseases, consisting of primary biliary cholangitis (PBC), primary sclerosing cholangitis (PSC), autoimmune hepatitis (AIH) and overlap variants. AIH and PBC are characterized by the presence of autoantibodies and elevated total immunoglobulin (Ig) G and IgM, respectively (1). The presence of autoantibodies against for example smooth muscle (SMA) and mitochondria (AMA) play an important role in the diagnostic scoring of AIH and PBC, respectively (2, 3). Although testing for different autoantibodies is implemented in the standard diagnostic work-up for liver disease with an unknown origin, they are not disease specific (4).

In another autoimmune disease, namely rheumatoid arthritis (RA), autoantibodies are also present, but in this disease antibodies frequently target proteins that have undergone post-translational modifications (PTM) (5). In particular, antibodies that target citrullination (anti-citrullinated antibodies: ACPA) and anti-carbamylated protein (anti-CarP) antibodies are used as diagnostic and prognostic markers in RA, respectively (6, 7). During inflammation, peptidyl arginine deiminases and cyanate are formed resulting in extracellular citrullination of arginine and carbamylation of lysine amino acids, respectively (8, 9). More recently, we have discovered antibody responses against the modifications malondialdehyde-acetaldehyde adducts (MAA) and advanced glycation end-products (AGE) in patients with systemic lupus erythematosus (SLE), defining a group of patients with neuropsychiatric manifestations (10). Both MAA and AGE are a result of oxidative stress and modify lysine amino acids (11, 12). Additionally, under oxidative stress nitration (NT) of the tyrosine amino acids and acetylation (AL) of lysines occur as a result of a reaction with peroxynitrite species and dysregulation of acetylation and deacetylation pathways, respectively (13, 14).

Inflammation occurs in both AIH and cholestatic liver disease, albeit at different sites (hepatocytes versus biliary tract). Oxidative stress occurs more frequently in patients with AIH compared to patients with cholestatic liver disease (15, 16). PTMs that are the result of oxidative stress have been reported to be highly immunogenic which could therefore result in anti-PTM antibody production, also in the context of AILD (17–19). However, studies assessing anti-PTM antibody responses in AILD are limited. Antibodies against cyclic citrullinated peptide (CCP) have been studied and were found in 9–11% of patients with type 1 AIH (20, 21), commonly in the absence of RA (21). Additionally, MAA modifications have shown to induce liver damage and to cause an autoimmune like pathophysiology in mice (22).

Since AIH, PBC and PSC are often considered (auto)immune mediated diseases that, like RA and SLE, display a variety of autoantibodies, we hypothesized that anti-PTM antibodies may be present in AILD and could have diagnostic or prognostic associations.

Here we report that anti-PTM antibodies are present in AILD, allow discrimination between subgroups of AILD and are related to treatment response in AIH.

## 2. Materials and methods

### 2.1. Study design and population

Patients visiting the Department of Gastroenterology and Hepatology of the Leiden University Medical Centre (LUMC) between 1996 and 2020 who signed informed consent for the Biobanking facility were eligible for inclusion. Patients visiting the Department of Gastroenterology and Hepatology of Erasmus Medical Centre, Rotterdam, with no objection against the use of residual material, were also included. The biobank protocol (B21.032) was prospectively approved by the Medical Ethical Committee of the LUMC. For the purpose of this study, patients were divided into three groups: AILD, (i.e., AIH, PBC or PSC), miscellaneous chronic liver diseases (non-AILD) and healthy controls (HC). HC were preselected from a biobank containing serum from healthy individuals. They were matched based on sex and age to the AILD cohort. No data on medical history of medication use was available, mimicking the general population. Although clinical, biochemical and histological overlap can occur, patients with overlap variants (AIH-PBC or AIH-PSC) were not included in the AILD cohort. AIH was diagnosed using the *revised original* or *simplified* criteria for the diagnosis for AIH (2, 23, 24). Patients with AIH were included at diagnosis. Of all AILD patients, 66 were diagnosed with AIH. Of these patients 8 patients already started treatment before inclusion. PBC and PSC were diagnosed according to the diagnostic criteria in the European guidelines and were included during follow-up (25). As the AIH cohort was the largest cohort with complete data, this was the cohort in which the final analyses were done.

### 2.2. Patient characteristics

Demographics and patient characteristics were collected from electronic patient files at the time of visit to the outpatient clinic. This included: age, sex, comorbidities, disease duration, presence of liver cirrhosis, simplified criteria for the diagnosis of AIH (24), revised original criteria for AIH (23), presence of self-reported arthralgia (i.e., extrahepatic manifestation of AIH) and medication use. Follow-up data (i.e., time to complete biochemical response (CBR), treatment response, mortality and liver transplantation) was also collected.

CBR was defined as normalization of aminotransferases and IgG below the upper limit of normal (26). Time to CBR was defined as the time from treatment initiation until the first time CBR was reached.

In addition to routine laboratory assessments (aspartate aminotransferase (ASAT), alanine aminotransferase (ALAT), IgG, gamma-glutamyl transferase (GGT), alkaline phosphatase (AP) and presence of autoantibodies), serum samples from each patient were collected (Supplementary Table S1). For patients with cholestatic liver disease, data regarding cholangiographic findings and laboratory assessments (GGT, AP and autoantibodies) were also collected (Supplementary Table S1).

### 2.3. Generation of PTMs

Modified proteins and their corresponding control non-modified protein were produced by either enzymatic or chemical reactions as previously described (10).

### 2.4. Assessment of anti-PTM antibodies

Anti-PTM antibodies were detected using an in-house enzyme-linked immunosorbent assay (ELISA), based on modified fetal calf serum (FCS) as described previously (10). Briefly, modified and non-modified FCS were coated to a Nunc Maxisorp ELISA plate (430,341, Thermofisher). In between each sequential step plates were washed three times using Phosphate Buffered Saline (PBS)/0.05%Tween (Sigma, P1379). After blocking [PBS/1% Bovine Serum Albumin (BSA)] for 6 h at 4°C, plates were incubated overnight at 4°C with 1/50, 1/100 or 1/1000 diluted serum. Each plate contained a standard of anti-PTM antibody positive serum to calculate arbitrary units. After incubation, IgG levels were detected using horseradish peroxidase (HRP) labeled Rabbit-anti-Human IgG (Dako, P0214). Plates were developed by incubating with 2,2′-azino-bis[3-ethylbenzothiazoline-6-sulfonic acid] (ABTS)/0.015% H_2_O_2_ (A1888 and 7,722-84-1, both from Merck) and absorbance at 415 nm was measured using a microplate reader (Bio-Rad iMark). The cut-off for positivity was set as the mean arbitrary units plus two times the standard deviation of 100 HCs, excluding values higher than 10x the mean.

### 2.5. Statistical analysis

Statistical analyses were performed using IBM SPSS 25.0 (IBM, Armonk, NY). Baseline characteristics were evaluated using descriptive statistics. Differences in levels of anti-PTM antibodies between HCs, AILD, and non-AILD were assessed using Kruskall-Wallis Test and Chi-2 test. Analyses of correlation between anti-PTM antibody levels and clinical variables were done using Spearman rank analyses for continuous clinical variables and point biserial correlation (i.e., mathematical equivalent of Pearson correlation) for dichotomous clinical variables. The anti-PTM antibody levels were transformed to natural logarithms to perform point-biserial correlations. Wilcoxon signed-rank test was used to compare anti-PTM antibody levels at baseline versus levels at the second visit.

Correlations between the difference in anti-PTM antibody levels at baseline versus the second visit and the change in levels of ALAT, ASAT and IgG were done using Spearman’s rho (r_s_). Landmark analysis was used for the evaluation of CBR, with pre-determined timepoints at 3, 6 and 12 months, to prevent immortal time bias. A *p*-value of <0.05 was considered statistically significant.

## 3. Results

### 3.1. Study cohort

We studied 207 patients with liver disease comprising an AILD cohort (*n* = 106) and a non-AILD cohort (*n* = 101). The AILD cohort consisted of patients with AIH (*n* = 66), PBC (*n* = 10) and PSC (*n* = 30) and was subsequently divided into two separate cohorts: AIH and cholestatic liver disease (CLD) (i.e., PBC and PSC). The non-AILD cohort consisted of patients with alcoholic liver disease (ALD) (*n* = 29), chronic hepatis B (HBV) (*n* = 4), chronic hepatitis C (HCV) (*n* = 22), non-alcoholic fatty liver disease (NAFLD) (*n* = 30), non-alcoholic steatohepatitis (NASH) (*n* = 1), or a combination of these (*n* = 15) (Table 1). In the AILD and non-AILD cohort 63.2 and 33.7% of the patients were female (*p* < 0.001) with a mean age of 48.2 ± 16.6 years and 54.0 ± 11.0 years, respectively (*p* = 0.003) (Table 1). Cirrhosis was present in 39.6% of the AILD cohort and in 56.4% of non-AILD patients (*p* = 0.035). In the AILD cohort, 96.7% of patients with PSC had large duct PSC on cholangiographic imaging. Eighty percent of PBC patients was AMA positive. The mean age of the HCs was 50.2 ± 10.5 years and 49% were female.

**Table 1:** Characteristics of study population with autoimmune mediated and cholestatic liver disease (AILD) and non-AILD at time of inclusion.

Patient characteristics	Auto-immune liver disease (AILD) (*n* = 106)	Non-autoimmune liver disease (non-AILD) (*n* = 101)	*p* value
Primary diagnosis			
*AIH*	66 (62.3)	-	
*PBC*	10 (9.4)	-	
*PSC*	30 (28.3)	-	
*NAFLD*	-	30 (29.7)	
*ALD*	-	29 (28.7)	
*HCV*	-	22 (21.8)	
*HBV*	-	4 (4.0)	
*NASH*	-	1 (1.0)	
*Hemochromatosis*	-	0 (0.0)	
*Combination*^†^	-	15 (14.9)	
Female sex	67 (63.2)	34 (33.7)	<0.001*
Age sample (years)	48.2 ± 16.6	54.0 ± 11.00	0.003*
Cirrhosis	42 (39.6)	57 (56.4)	0.035*
*Yes, compensated*	28 (26.4)	27 (26.7)	-
*Yes, decompensated*	14 (13.2)	30 (29.7)	-
*No cirrhosis*	59 (55.7)	44 (43.6)	-
*Unknown*	5 (4.7)	0 (0.0)	-

Results are presented as *n* (%), mean ± SD or median (IQR). *p* < 0.05 is considered statistically significant (*). AIH, autoimmune hepatitis; AILD, autoimmune liver disease; ALD, alcoholic liver disease; HBV, chronic hepatis B; HCV, chronic hepatitis C; NAFLD, non-alcoholic fatty liver disease; NASH, non-alcoholic steatohepatitis; PBC, primary biliary cholangitis; PSC, primary sclerosing cholangitis. † Combinations: ALD + HBV (*n* = 1), ALD + HCV + HBV (*n* = 1), ALD + HCV (*n* = 6), ALD and hemochromatosis (*n* = 2), ALD + NASH (*n* = 2), ALD + PSC (*n* = 1), HBV + HDV (*n* = 1), HCV + HIV (*n* = 1).

### 3.2. Anti-MAA, anti-AGE, anti-CarP, and anti-AL antibodies are more prevalent in AILD compared to HC and non-AILD and are more likely to be positive for more than one anti-PTM antibody

**Figure 1:**
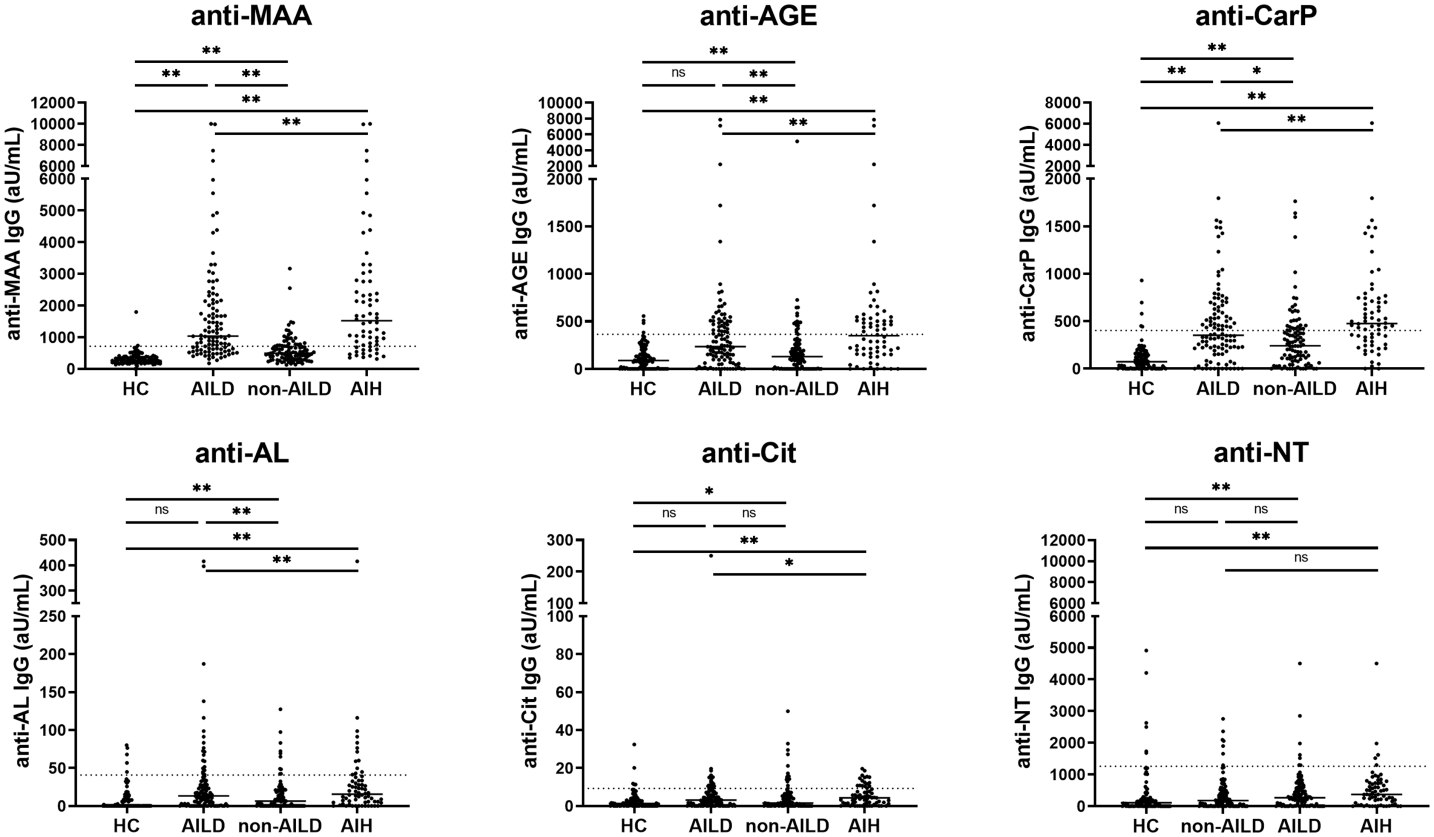
Anti-MAA, anti-AGE, anti-CarP, and anti-AL antibodies are increased in patients with AILD, and especially in patients with AIH. IgG antibody levels are presented as arbitrary units per milliliter (aU/mL) and cut-off for each PTM is indicated by the dashed line. *p < 0.05, **p < 0.01. Autoimmune Liver Disease: AIH, PBC and PSC; non-Autoimmune Liver Disease: NAFLD, HCV, HBV, ALD, Combination, NASH. AGE, advanced glycation end-product; AIH, autoimmune hepatitis; AL, acetylated protein; CarP, carbamylated protein; Cit, citrullinated protein; MAA, malondialdehyde–acetaldehyde adduct; ns, not significant; NT, nitrated protein.

Anti-PTM IgG antibody levels directed against 6 PTMs were measured in 207 patients with liver disease and 100 HCs (Figure 1 *and* Supplementary Table S2). Anti-MAA, anti-AGE, anti-CarP and anti-AL antibody levels differed significantly between AILD and HCs (1036.0, 234.5, 352.5 and 13.3 aU/mL vs. 266.9, 88.9, 74.0 and 0.0 aU/mL respectively, all *p* < 0.01). Only anti-MAA and anti-CarP antibodies were significantly increased when comparing non-AILD to HCs (495.8 and 241.0 aU/mL vs. 266.9 and 74.0 aU/mL, respectively, both *p* < 0.01). Additionally, AILD showed significantly higher median levels of anti-MAA, anti-AGE, anti-CarP and anti-AL antibodies compared to non-AILD (anti-MAA, anti-AGE and anti-AL 1036.0, 234.5, 13.3 aU/mL vs. 495.8, 130.0, 6.4 aU/mL, respectively, *p* < 0.01 and anti-CarP 352.5aU/mL vs. 241.0 aU/mL, *p* < 0.05). Median levels of anti-NT and anti-Cit differed significantly between AILD and HCs (269.0 and 3.1 aU/mL vs. 108.0 and 1.3 aU/mL, *p* < 0.01 and *p* < 0.05, respectively) but did not differ significantly between non-AILD and HCs.

**Figure 2:**
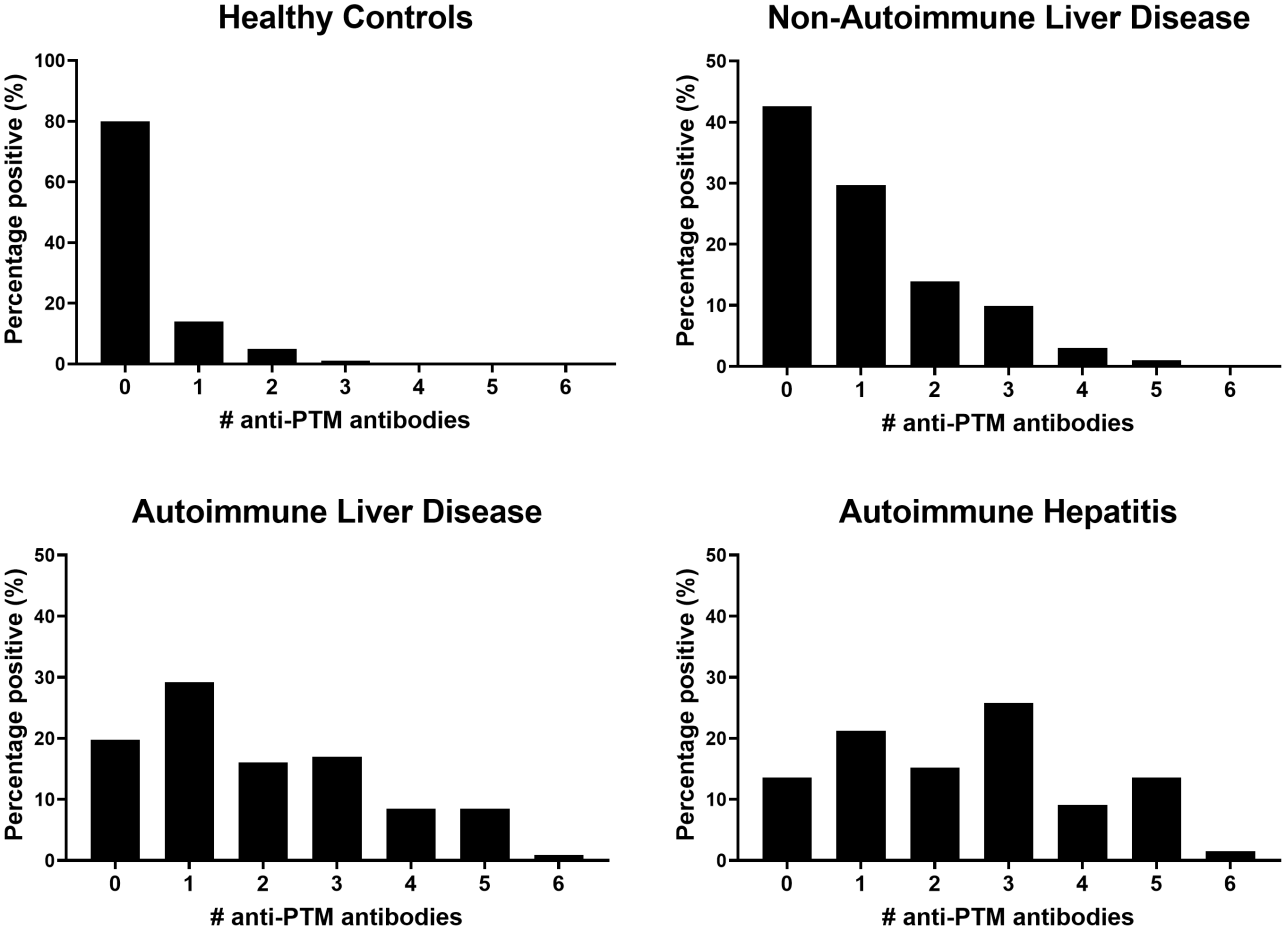
Patients with AILD, and especially AIH, are more likely to be positive for more than one anti-PTM antibody. Data is presented as percentage positive (%) patients for a number of anti-PTM antibodies in (from left to right) healthy controls, non-autoimmune liver disease autoimmune liver disease and autoimmune hepatitis.

Comparing the frequency of positivity, AILD patients showed significantly increased positivity of anti-MAA, anti-AGE, anti-CarP and anti-AL antibodies compared to HCs (67.9, 36.8, 47.2 and 18.9% vs. 2.0, 4.0, 5.0, and 5.0%, all *p* < 0.01). Increased positivity for anti-MAA, anti-AGE and anti-CarP antibodies (28.7, 17.8 and 27.7% vs. 2.0, 4.0 and 5.0%, respectively, all *p* < 0.01) was observed when comparing non-AILD and HCs. Additionally, increased positivity between non-AILD and AILD was observed for anti-MAA, anti-AGE and anti-CarP antibodies (67.9, 36.8 and 47.2% vs. 26.7, 17.8 and 27.7%, respectively, all p < 0.01). Also when the non-AILD control group is limited to a more stringent set of conditions, excluding HBV, NASH and hemochromatosis, all statistical associations remain intact (data not shown). Anti-PTM antibody positivity for different anti-PTM antibodies were combined to calculate positivity for multiple anti-PTM antibodies (Figure 2). Patients with AILD more frequently harbored at least one type of anti-PTM antibody compared to non-AILD and HCs (AILD: 81.2%, non-AILD: 58.4% and HCs: 20%). The data in Figure 2 also indicate that AILD patients are more likely to be positive for multiple anti-PTM antibodies. Overall, these data indicate that anti-PTM antibodies are especially present in patients with AILD.

### 3.3. Within AILD, patients with AIH harbor anti-PTM antibodies more often and present with specific combinations of anti-PTM antibodies

Next, AILD was dissected into the three major immune liver disease subgroups, namely AIH, PBC and PSC. Presence of anti-MAA, anti-AGE, anti-CarP and anti-AL antibodies were assessed in these subgroups, or AIH alone, and compared to non-AILD (Figures 1, 2, and Supplementary Table S3). Interestingly, patients with AIH harbored significantly more of these antibodies compared to non-AILD patients (anti-MAA: 77.3% vs. 26.7%, anti-AGE: 48.5% vs. 17.8% and anti-CarP: 63.6% vs. 27.7%, all *p* < 0.001, respectively). Within the AILD cohort, predominantly patients with AIH harbored anti-PTM antibodies. We did however also see some patients with CLD who were positive for some. Subsequently, the AILD was divided into two cohorts: AIH and CLD. Patients with AIH were significantly more often positive for anti-MAA, anti-AGE, anti-CarP and anti-Cit (77.3, 48.5, 63.6 and 25.8% vs. 52.5, 17.5, 20.0 and 2.5% respectively, all *p* < 0.01) compared to patients with CLD (Table 2).

**Table 2:** The association between the presence of anti-PTM antibodies in HC, non-AILD, AIH and cholestatic liver disease.

	Healthy controls *n* = 100				Non-autoimmune liver disease *n* = 101				Autoimmune hepatitis *n* = 66				Cholestatic liver disease *n* = 40			
	aU/mL [IQR]		*n* (% positive)		aU/mL [IQR]		*n* (% positive)		aU/mL [IQR]		*n* (% positive)		aU/mL [IQR]		*n* (% positive)	
*Anti-MAA*	266.9	[200.4–370.2]	2	(2.0)	495.8	[315.2–726.8]	27	(26.7)	1519.5	[760.0–2775.3]	51	(77.3)	771.5	[538.3–1247.7]**,#	21	(52.5)**,#,+
*Anti-AGE*	88.9	[0.0–182.5]	4	(4.0)	130.0	[4.2 –261.2]	18	(17.8)	349.0	[156.0–537.0]	32	(48.5)	143.5	[27.0–304.0]+	7	(17.5)*,+
*Anti-CarP*	74.0	[1.5–157.9]	5	(5.0)	241.0	[83.5 –422.0]	28	(27.7)	475.5	[293.2–741.8]	42	(63.6)	226.5	[9.8–328.5]**,++	8	(20.0)*,++
*Anti-AL*	0.0	[0.0–9.8]	5	(5.0)	6.4	[0.0 –10.0]	10	(9.9)	15.4	[4.7–33.4]	13	(19.7)	10.8	[0.5–25.4]*	7	(17.5)*
*Anti-Cit*	1.3	[0.0–3.2]	6	(6.0)	1.6	[0.0 –5.9]	15	(14.9)	4.3	[1.2–9.7]	17	(25.8)	1.8	[0.0–4.2]	1	(2.5)#,+
*Anti-NT*	108.0	[0.0–250]	6	(6.0)	179	[0.0–501.5]	7	(6.9)	369.0	[65.8–732.5]	5	(7.6)	212.0	[0.0–400.8]	2	(5.0)

Results are presented as median (IQR) of *n* (%). Chi-2-tests were used to assess the difference between the presence of the specific manifestations and non-AILD patients. HC versus cholestatic liver disease *p < 0.005, **p < 0.001; non-AILD versus cholestatic liver disease #*p* < 0.005, ##*p* < 0.001; and AIH versus Cholestatic liver disease +*p* < 0.005, ++*p* < 0.001. AGE, advanced glycation end-product; AIH, autoimmune hepatitis; AL, acetylated protein; aU/mL, arbitrary units per milliliter; CarP, carbamylated protein; Cit, citrullinated protein; IQR, interquartile range; MAA, malondialdehyde–acetaldehyde adduct; NT, nitrated protein; non-AILD, non-autoimmune liver disease; PBC, Primary Biliary Cirrhosis; PSC, Primary Sclerosing Cholangitis.

Analysis of different anti-PTM antibody combinations showed that AILD patients mostly harbored a combination of anti-MAA, anti-AGE and anti-CarP antibodies (15/85 = 17.6%) or anti-MAA and anti-CarP antibodies (7/85 = 8.2%) compared to non-AILD (anti-MAA/-AGE/-CarP: 5/58 = 8.6% and anti-MAA/-AGE/-CarP: 3/58 = 5.2%) (Supplementary Figures S1A,B). Strikingly, comparing AIH patients with total AILD, all double (anti-MAA/-AGE/-CarP), almost all (except 1) triple (anti-MAA/-AGE/-CarP) and all quintuple (anti-MAA/-AGE/-CarP/-AL/-Cit) positive patients from the AILD group belonged to the AIH group (Supplementary Figure S1C). Taken together, patients with AIH harbored anti-PTM antibodies more often compared to other subgroups of AILD.

### 3.4. There are no significant associations between anti-PTM antibody positivity, presence of ANA and SMA, cirrhosis and sex in AIH patients

In AIH several other antibodies have been described such as ANA and SMA. We have analyzed to what extent these antibodies occur together with the anti-PTM antibody responses or to what degree detectable anti-PTM antibody responses differ depending on the positivity status for ANA or SMA. We did not observe a significant difference in the presence of anti-PTM antibodies in patients positive or negative for ANA or SMA, with the exception of anti-MAA positivity and ANA positivity in patients with AIH (Chi-2 (1) > = 4.687, *p* = 0.030). We further analyzed the positivity for anti-PTM antibodies in patients with AIH who were negative for both ANA and SMA. Despite it being a small cohort (n = 11), we observed that the absolute percentages for positivity of anti-MAA, anti-AGE, anti-CarP, anti-AL, anti-Cit and anti-NT was in general higher in patients who were both ANA and SMA negative compared to patients who were either ANA negative of SMA negative (Table 3). This further supports the idea that anti-PTM antibodies provide different information compared to the already known antibodies ANA and SMA. Additionally, in the AIH cohort positivity for any of the anti-PTM antibodies did not show significant differences between patients when stratifying for cirrhosis. Furthermore, in the AILD cohort, we observed a significant association between anti-CarP and female sex (Chi-2 (1) > = 4.740, *p* = 0.029). In the AIH cohort however, none of the anti-PTM antibodies showed significant associations with sex.

**Table 3:** Percentage of positivity for anti-PTM antibodies in ANA positive, ANA negative, SMA positive, SMA negative and double negative (ANA and SMA) patients with AIH.

	AIH (*n* = 66)				
	ANA positive (*n* = 42)	ANA negative (*n* = 24)	SMA positive (*n* = 35)	SMA negative (*n* = 31)	ANA negative / SMA negative (*n* = 11)
Anti-MAA positive	85.7%	62.5%	71.4%	83.9%	81.8%
Anti-AGE positive	54.8%	37.5%	40.0%	58.1%	63.6%
Anti-CarP positive	69.0%	54.2%	62.9%	64.5%	72.7%
Anti-AL positive	23.8%	12.5%	20.0%	19.4%	27.3%
Anti-Cit positive	31.0%	16.7%	22.9%	29.0%	27.3%
Anti-NT positive	4.8%	12.5%	8.6%	6.5%	18.2%

AGE, advanced glycation end-product; AL, acetylated protein; ANA, anti-nuclear antibodies; CarP, carbamylated protein; Cit, citrullinated protein; MAA, malondialdehyde–acetaldehyde adduct; NT, nitrated protein; SMA, smooth muscle antibody.

### 3.5. Anti-MAA and anti-CarP antibodies significantly correlate with measures of biochemical treatment response

**Figure 3:**
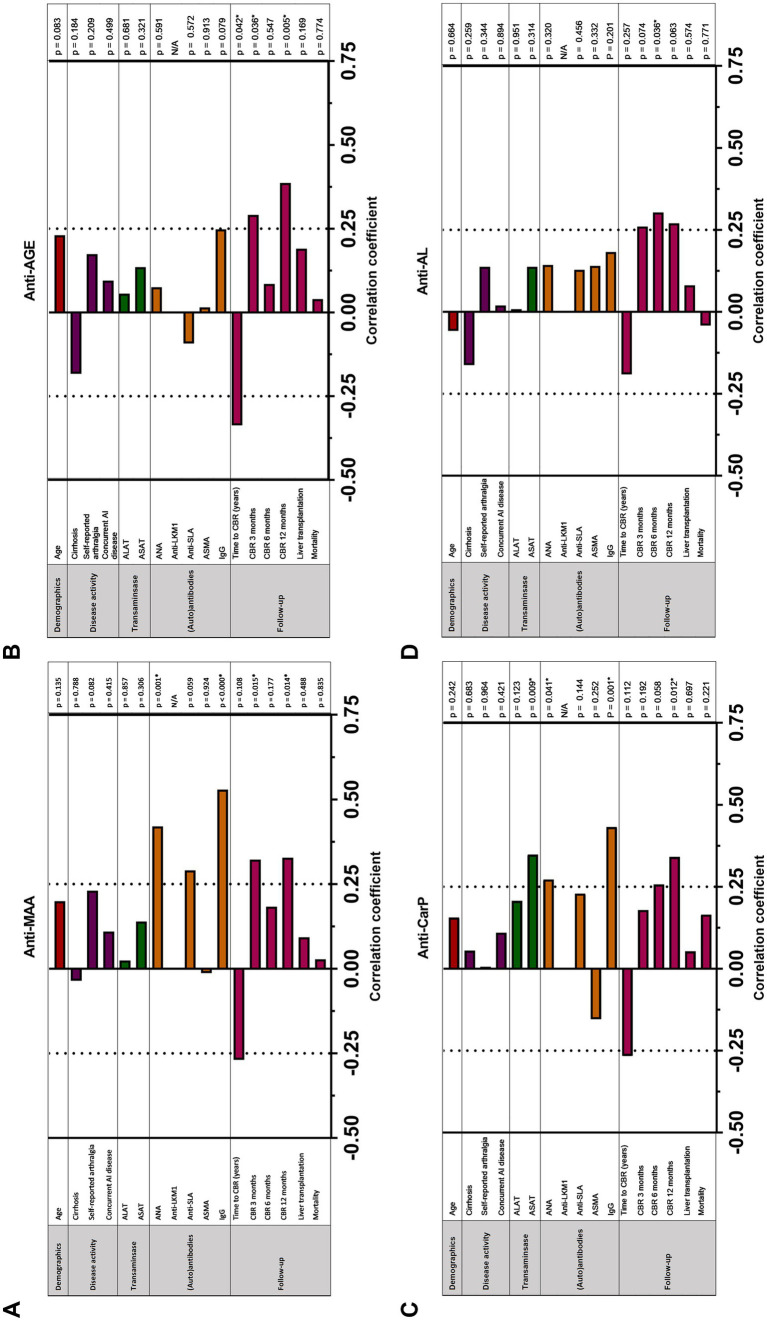
Correlation between **(A)** anti-MAA IgG, **(B)** anti-AGE IgG, **(C)** anti-CarP IgG and **(D)** anti-AL IgG antibodies and clinical and serological markers in patients with untreated auto-immune hepatitis (*n* = 58). Correlation analyses are done using Spearman’s rho correlation analysis and point-biserial correlation analysis. *p* < 0.05 is considered statistically significant (*). AGE, advanced glycation end-product; ALAT, alanine aminotransferase; ANA, anti-nuclear antibodies; ASAT, aspartate aminotransferase; SMA, smooth muscle antibody; CBR, complete biochemical response; IgG, immunoglobulin gamma; LKM, Liver Kidney microsomal antibody; SLA, soluble liver antigen.

We investigated if increased anti-PTM antibodies correlated with commonly used serological and clinical markers in patients with AIH (Figures 3A–D and Supplementary Table S4). As treatment for AIH consists of immunomodulatory treatment, and might therefore influence biochemical markers, only patients with treatment naïve AIH were included in these analyses (Table 4). Both anti-MAA and anti-CarP correlated positively with serum IgG (*p* < 0.000/*p* = 0.001) and antinuclear antibodies (ANA) (*p* = 0.001). Anti-CarP correlated positively with ASAT (*p* = 0.009). We demonstrated correlations between anti-MAA, self-reported arthralgia and antibodies against soluble liver antigen (SLA) approaching statistical significance (*p* = 0.082 and 0.059 respectively). No significant correlations were found for anti-AGE and anti-AL.

**Table 4:** Characteristics of untreated AIH patients in the AILD cohort (*n* = 58).

Patient characteristics	Autoimmune hepatitis (*n* = 58)
Female sex	43 (74.1)
Age diagnosis (years)	46.4 ± 19.4
Simplified criteria for the diagnosis of AIH	8 (6–8)
Original revised criteria for AIH	16.7 ± 3.3
Positive antibodies	
*ANA (n = 58)*	38 (65.5)
*SMA (n = 58)*	29 (50.0)
*Anti-LKM (n = 43)*	0 (0.0)
*Anti-SLA*	2 (3.4)
Others*	8 (13.8)
IgG (*n* = 57)	24.80 (19.85–32.65)
Histology	
*Typical*	45 (77.6)
*Compatible*	9 (15.5)
*Atypical/biopsy not done*	3 (5.2)
Negative viral hepatitis serology	57 (98.3)
Cirrhosis	23 (39.7)
*Yes, compensated*	14 (24.1)
*Yes, decompensated*	9 (15.5)
No cirrhosis	35 (60.3)
Unknown	0 (0.0)
Self-reported arthralgia	12 (20.7)
(More than one) concomitant auto immune disease**	17 (29.3)

Results are presented as *n* (%), mean ± SD or median (IQR). AIH, autoimmune hepatitis; ANA, anti-nuclear antibodies; SMA, smooth muscle antibody; IgG, immunoglobulin gamma; IQR, interquartile range; LKM, Liver Kidney microsomal antibody; SD, standard deviation; SLA, soluble liver antigen. *Others: pANCA (*n* = 8) **Other: auto-immune hemolysis (*n* = 1), celiac disease (*n* = 1), diabetes mellitus type 1 (*n* = 2), granulomatosis with polyangiitis (*n* = 1), Henloch-Schönlein purpura (*n* = 1), Hyperthyroidism (*n* = 3), hypothyroidism (*n* = 6), myastenia gravis (*n* = 1), sclerodermia (*n* = 2) ulcerative colitis (*n* = 1).

### 3.6. Anti-MAA, anti-AGE, and anti-CarP antibodies positively correlate with CBR

Time to CBR negatively correlated with the presence of anti-PTM antibodies in patients with AIH, reaching significance for anti-AGE (*p* = 0.042) (Figures 3A–D). In line with these findings, anti-MAA and anti-AGE correlated positively with CBR at 3 months (*p* = 0.015 and 0.036, respectively). In addition, anti-MAA, anti-AGE, and anti-CarP positively correlated with CBR at 12 months (*p* = 0.014, 0.005, and 0.012, respectively) (Figures 3A-D). A trend toward significance was found for anti-AL and CBR at 12 months. No association between the presence of anti-PTM antibodies and long-term follow-up (i.e., liver transplantation or mortality) was found. A logistic regression was performed to analyze the effects of positivity for all six individual anti-PTM antibodies on the likelihood of reaching CBR at 3, 6 and 12 months. Positivity for any individual anti-PTM antibody was not independently associated with an increased or decreased likelihood of reaching CBR at 3, 6 or 12 months (data not shown).

### 3.7. Patients with AIH and positive for at least three anti-PTM antibodies reach CBR quicker after initiating treatment

Based on the discovery of multiple anti-PTM antibody positivity in patients with AIH, we attempted to discover the clinical relevance of harboring these multiple anti-PTM antibodies. The median follow-up was 8.7 years (4.6–15.3) (Supplementary Table S5).

Patients with at least three anti-PTM antibodies scored significantly higher on the revised original score for AIH and had significantly higher levels of IgG at time of diagnosis (Supplementary Table S5). Aminotransferase levels were higher in the group with at least three positive anti-PTM antibodies, albeit not significant. Anti-MAA and anti-CarP correlated positively with ASAT at baseline in the group with less than three anti-PTM antibodies present (r_s_ = 0.37 and r_s_ = 0.45, *p* = 0.037 and *p* = 0.009 respectively), but not in AIH patients with at least three anti-PTM antibodies. After 3 months treatment, significantly more AIH patients with at least three anti-PTM antibodies had reached CBR (*p* = 0.03). After 12 months of treatment, the difference was still significant (*p* = 0.01). Overall, a trend toward significance for time to CBR (in years) was found in favor of multiple anti-PTM antibody positivity.

### 3.8. Anti-PTM antibody responses decrease over time and show distinct associations with ALAT, ASAT or total IgG levels.

**Figure 4:**
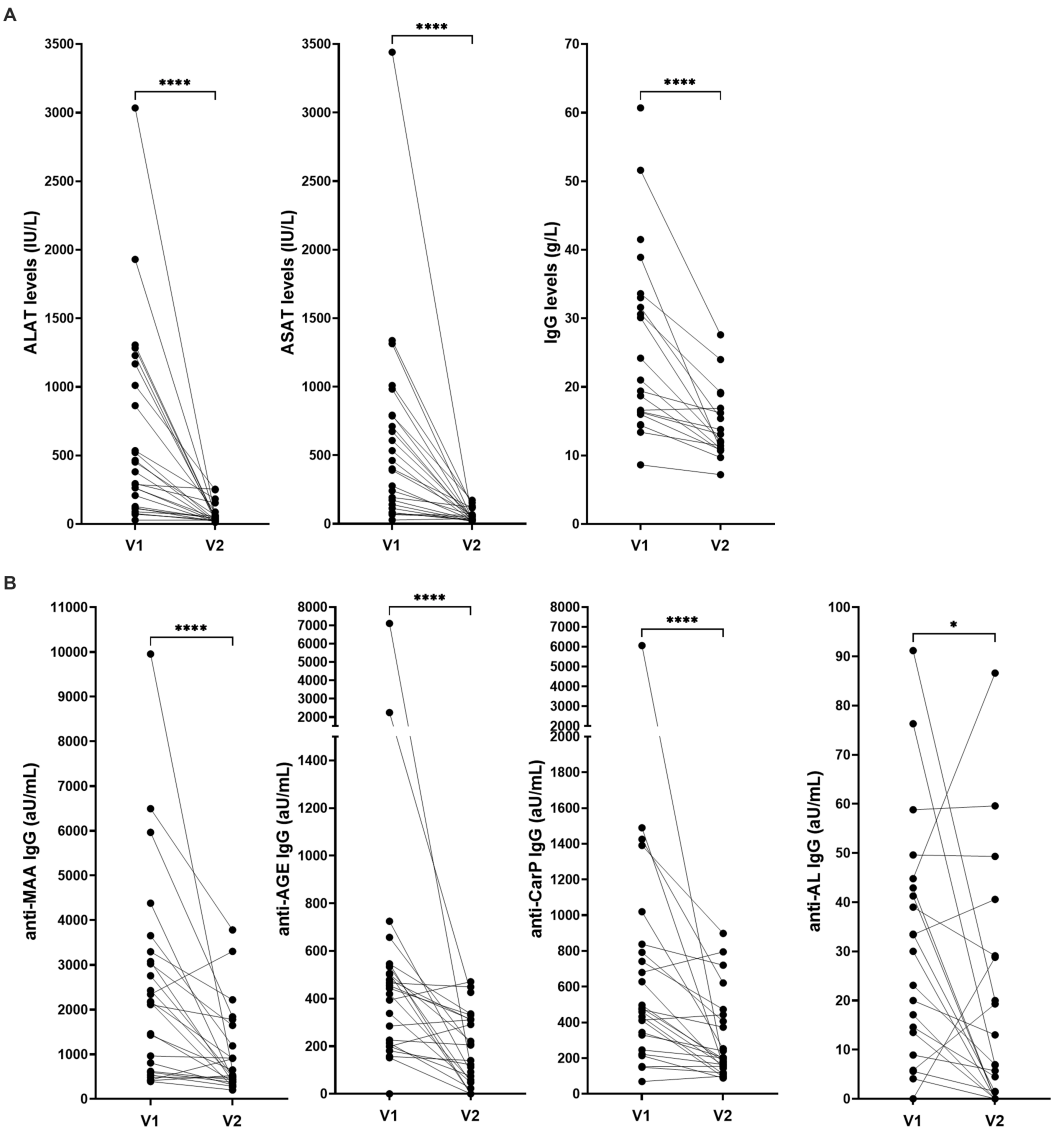
Levels of **(A)** ALAT, ASAT, IgG and **(B)** anti-PTM antibodies over time in patients with AIH (*n* = 25). ALAT, ASAT and IgG levels were determined as standard procedure after inclusion. V1: before commencing treatment, V2: during treatment. Median time interval: 65 months (6–138). Reactivity toward anti-PTM antibodies was determined using ELISA and is depicted as arbitrary units per milliliter (aU/mL). *p = 0.0244 and ****p ≤ 0.0001. AGE, advanced glycation end-product; AL, acetylated protein; ALAT, Alanine aminotransferase; ASAT, Aspartate aminotransferase; CarP, carbamylated protein; IgG, immunoglobulin gamma; MAA, malondialdehyde–acetaldehyde adduct; V1, first visit; V2, second visit.

Clinical data of two different timepoints were available of 25 AIH patients and antibody responses over time was investigated (Figure 4). The first sample was taken before commencing treatment, the second sample during treatment. The median time interval between visit one and two was 65 months (6–138). Median ΔALAT, ΔASAT and ΔIgG were 352 IU/L (951–79), 324 IU/L (722–83) and 8 g/L (14–2) respectively. Levels of all four anti-PTM antibody responses decreased significantly over time (anti-MAA, anti-CarP, and anti-AL (*p* ≤ 0.0001) and anti-AGE (*p* = 0.024)). Change in anti-AL antibody titers associated significantly with change in ASAT and ALAT (r_s_: 0.46 and 0.40 *p* = 0.02 and 0.05 respectively) but did not associate with change in total IgG (r_s_: 0.17 *p* = 0.53) (Supplementary Table S6). Change in anti-AGE antibody titers significantly associated with change in IgG (r_s_: 0.63 *p* = 0.007). Change in anti-MAA and anti-CarP antibody levels was not associated with decrease in ALAT, ASAT and IgG. However, change in anti-CarP antibody levels did show a positive trend toward significant association with decrease of IgG (r_s_: 0.48 *p* = 0.052) (Supplementary Table S6).

## 4. Discussion

To the best of our knowledge, this is the first report on the presence of anti-PTM antibodies in AILD. The presence of anti-PTM antibodies has been described in several other autoimmune diseases where they can serve as diagnostic or prognostic markers (6, 7, 27). Based on these results, we hypothesized that anti-PTM antibodies are also generated in patients with AILD. Additionally, we speculated that patterns in the presence of anti-PTM antibodies might serve diagnostic or prognostic purposes in AILD.

In this study there were five significant findings: First, four anti-PTM antibodies were more prevalent in patients with AILD compared to HCs and to non-AILD: anti-MAA, anti-AGE, anti-CarP, and anti-AL. Second, patients with AILD and particularly patients with AIH often harbored multiple types of anti-PTM antibodies. Third, anti-MAA and anti-CarP antibody positivity significantly correlated with markers for biochemical response in AIH. Fourth, AIH patients with at least three types of anti-PTM antibodies reached CBR at 12 months after initiating treatment more frequently. Lastly, after initiating immunosuppressive treatment next to aminotransferases and IgG also anti-AGE and anti-AL antibody titers decreased. These findings confirmed that anti-PTM antibodies are present in AILD and moreover multiple anti-PTM antibodies identify a group of AIH patients in which these anti-PTM antibodies associate with CBR. Interestingly, we observed that several anti-PTM antibodies are present and even more prevalent in AIH patients who were ‘sero-negative’ for the classical autoantibodies at diagnosis, compared to patients who were positive for either ANA or SMA. This is particularly captivating since conventional antibodies are not disease specific and may be expressed at a later stage of the disease in ‘sero-negative’ AIH patients. We suggest that anti-PTM antibodies may be present in patients with AIH before conventional antibodies can be detected. Therefore anti-PTM antibody assessment could especially be interesting in the diagnostic work-up for ‘sero-negative’ AIH patients. Future studies should further determine the possible implementation of anti-MAA, anti-AGE or anti-CarP assessment, all associated with CBR at 12 months, in the diagnostic algorithm for AIH.

The clinical presentation of AIH is very heterogeneous and can vary from asymptomatic disease to acute (on chronic) liver failure. Occasionally, polyarthralgia without arthritis is present in patients with AIH (1, 28), and is considered an extra hepatic manifestation of AIH. However, this is often not recognized and is underreported. In clinical practice, reoccurrence of arthralgia is often seen during corticosteroid withdrawal (28). Next to arthralgia, RA is sometimes seen in AIH. We have previously reported that, in the context of RA, anti-CarP (29) and anti-Cit (30) antibodies in arthralgia predict development of RA. Additionally, anti-PTM antibodies have been described in the context of rheumatic disease (10, 31, 32). In this study only a trend was found for the correlation between self-reported arthralgia and anti-MAA antibodies in AIH. This could be a result of the small cohort size and would require further investigation.

Previous research showed that IgG levels are not associated with long-term outcomes in AIH, whereas normalization of aminotransferases is the main treatment goal in AIH, as this positively associates with survival in the first 12 months after diagnosis (33). Additionally, Hartl et al. found that patients with normal IgG levels showed a comparable treatment response to patients with elevated IgG (34). On the contrary, CBR is defined as normalization of ALAT, ASAT and IgG (26). The role of IgG remains a pivoting point in disease progression in AIH. The results of this study suggest that specific subsets of anti-PTM antibodies are associated with treatment response.

In this study, patients with AIH positive for at least three types of anti-PTM antibodies had significantly higher IgG levels at diagnosis and tended to reach CBR more often at 12 months of treatment than patients with AIH with less than three anti-PTM antibodies. By choosing more than three anti-PTM reactivities as a cut-off in this analysis we achieved an equal number of AIH patients in each group (26 with less and 32 with at least three anti-PTM antibodies). Larger studies could determine whether combinations of anti-PTM antibodies, also combined with serum levels of IgG, ALAT, and ASAT at baseline could be better predictors for the likelihood of treatment response. The anti-PTM antibody response is an IgG mediated response and is part of the significantly elevated IgG in this specific group of patients. Positivity for multiple autoantibodies has been reported to provide more reliable information than single biomarkers in for example diabetes (35) and pre-RA (36).

Our study has some limitations: the cohort is heterogeneous and has a limited size. We found that 40 % of AIH patients identify with specific combinations of anti-PTM antibodies (anti-MAA/-AGE/-CarP; anti-MAA/-AGE/-CarP; anti-MAA/-AGE/-CarP/-AL/-Cit) within the AILD group. These specific combinations might aid in the diagnostic work-up for AIH. Noteworthy is that anti-PTM antibodies are not solely found in AIH, but are also found in other liver diseases possibly as a result of breach in tolerance against PTMs that are formed during inflammation. For several autoimmune diseases it is well known that certain autoantibodies are already present many years before the patients develop clinically overt disease for example anti-Cit and anti-CarP Ab in the context of RA (6, 7). Whether this is also the case in these autoimmune liver diseases is currently unknown. PTMs formed as a consequence of inflammation together with impaired liver function may well accumulate and mediate a breach in tolerance, and in this setting anti-PTM antibodies can be formed as a consequence of liver disease. The same set of 6 anti-PTMs were studied in SLE, and anti-MAA, anti-AGE and anti-CarP antibodies were also most frequently found in patients with SLE compared to healthy controls (10). Interestingly, anti-MAA and anti-CarP associated with neuropsychiatric manifestations of SLE, a manifestation that lacked a biomarker. These findings are in the same range as anti-PTM antibody responses found in AILD. Anti-CarP and anti-Cit are well studied anti-PTM antibodies in RA and are found in approximately 50% of RA patients (6, 7). Discovery of new anti-PTM antibodies in RA helped in diagnosis and in following disease progression, and can potentially help to distinguish groups within so-called seronegative RA (5). In order to further validate these findings and prove the sensitivity and specificity of these anti-PTM antibody combinations in the diagnostic work-up of AIH, anti-PTM antibodies need to be assessed extensively in a larger cohort. This could provide the opportunity to set a cut-off titer level and perhaps even distinguish AIH from other liver diseases. In this limited cohort it was not possible to evaluate the prognostic value of anti-PTM antibodies for disease progression as 40% of patients already had cirrhosis at diagnosis. A larger cohort study should be conducted in patients with AILD and no cirrhosis at diagnosis with set follow-up timepoints.

Different clinical parameters are measured to monitor disease activity for AIH, PBC and PSC. As a result, the three groups within the AILD cohort are incomparable. However, in the AILD cohort we have included PBC and PSC, which are not pure auto-immune diseases (where immune injury results in cholestasis) (25, 37–43). We hoped to evaluate possible differences in anti-PTM antibodies patterns in AIH, PBC and PSC. When analyzing AIH patients, only untreated patients were included to prevent impact of treatment. One of the strengths of this study is that the group of interest, AILD, is well-defined according to simplified or original revised score for AIH. Therefore, we can state that the results found for these subgroups are representative.

Combining the prevalence data of all six anti-PTM antibodies tested we observe that approximately 20% of healthy controls harbored at least one anti-PTM antibody (Figure 2). PTM of proteins occurs in all individuals, these PTMs may represent neo-epitopes toward which antibodies can be formed. Interestingly, this is apparently often not associated with disease, but is known to predispose to disease.

The standard therapy for patients with AIH consists of a combination of glucocorticoids and azathioprine (2). Most patients with AIH in this cohort were initially treated with this preferred treatment. Pape et al. demonstrated that a higher or lower initial predniso(lo)ne dose does not have impact on reaching CBR. In our study, stratification of the results by the initial steroid dose was not possible, as 42 patients (85.7%) received an initial predniso(lo)ne dose above 30 mg/day (44). When patients do not reach CBR, the treating physician may decide to intensify or adjust treatment regimens. The nature of the disease, characterized by intermittent loss of remission and flares, may give reason to frequently adjust therapy. The size of the studied cohort limited us to correct for change in therapy over time. Since it was not possible to obtain the necessary data we were not able to correct the correlation analyses regarding CBR for duration of steroid treatment, duration of tapering schemes, dose modification or drug withdrawal during follow-up. The median ASAT and ALAT did not differ between the patients who were prescribed budesonide compared to predniso(lo)ne, although this has been previously reported (45).

However, according to the guidelines and Delphi consensus on treatment response, treatment effect is first evaluated 6 months after commencing treatment (2, 26). We additionally did see more patients reaching CBR at 12 months of treatment if they had at least three positive anti-PTM antibodies. This may imply that having anti-PTM antibodies for at least three PTMs may be prognostically favorable regarding treatment response. Despite higher ALAT and ASAT levels at baseline in the AIH patients with at least three anti-PTM antibodies present, no association between transaminase levels and multiple positivity could be found. Only in patients with less than three anti-PTM antibodies present, a positive correlation between anti-MAA, anti-CarP and ASAT was found. This strengthens the implication that multiple positivity for at least three anti-PTM antibodies may be beneficial for treatment response and may guide treating physicians to earlier treatment intensification.

In conclusion, anti-PTM antibodies are present in patients with AILD. Some patients are positive for multiple anti-PTM antibodies. Having three or more anti-PTM antibody responses is associated with a favorable response to treatment in AIH.

## Data availability statement

The data analyzed in this study is subject to the following licenses/restrictions: Data, analytic methods, and study materials will be made available to other researchers upon any reasonable request. Requests to access these datasets should be directed to L.A.Trouw@lumc.nl.

## Author contributions

MB, AS, AM, SM, LZ, MT, BH, and LT were involved in the design and interpretation of the study. Anti-PTM antibody analyses were performed by MB under the supervision of LT. Clinical data was collected by AS under the supervision of BH and MT. Statistical analysis and interpretation were performed by MB and AS under the supervision of LT, BH, and MT. AM kindly provided cohort sera samples. MB and AS drafted the manuscript, which was critically revised by LT, BH, MT, AM, SM, and LZ. All authors contributed to the article and approved the submitted version.

## Funding

MB, SM, LZ, and LT have received funding from the European Research Council (ERC) under the European Union’s Horizon 2020 research and innovation program (grant agreement no 724517). AS, MT, and BH received a ZonMW grant (nr 10140022010001) and funding from Chiesi Pharmaceuticals BV (project number: PA 2019–71111) for a different project on the topic of autoimmune hepatitis.

## Conflict of interest

The authors declare that the research was conducted in the absence of any commercial or financial relationships that could be construed as a potential conflict of interest.

## Publisher’s note

All claims expressed in this article are solely those of the authors and do not necessarily represent those of their affiliated organizations, or those of the publisher, the editors and the reviewers. Any product that may be evaluated in this article, or claim that may be made by its manufacturer, is not guaranteed or endorsed by the publisher.

## Glossary

AILD: (auto)immune mediated and cholestatic liver disease
PBC: primary biliary cholangitis
PSC: primary sclerosing cholangitis
AIH: autoimmune hepatitis
Ig: immunoglobulin
SMA: smooth muscle antibodies
AMA: anti-mitochondrial antibody
RA: rheumatoid arthritis
PTM: post-translational modification
ACPA: anti-citrullinated antibodies
CarP: carbamylated protein
MAA: malondialdehyde-acetaldehyde adduct
AGE: advanced glycation end-products
SLE: systemic lupus erythematosus
NT: nitration
Anti-PTM antibody: anti-post-translationally modified protein antibody
CCP: cyclic citrullinated peptide
Non-AILD: non-autoimmune mediated and cholestatic liver disease
HC: healthy control
CBR: complete biochemical response
ASAT: aspartate aminotransferase
ALAT: alanine aminotransferase
ELISA: enzyme-linked immunosorbent assay
FCS: fetal calf serum
PBS: phosphate buffered saline
BSA: bovine serum albumin
HRP: horseradish peroxidase
ABTS: 2,2′-azino-bis(3-ethylbenzothiazoline-6-sulfonic acid)
AL: acetylation
ALD: alcoholic liver disease
HBV: chronic hepatis B
HCV: chronic hepatitis C
NAFLD: non-alcoholic fatty liver disease
NASH: non-alcoholic steatohepatitis
Cit: citrullination
ANA: antinuclear antibodies
SLA: soluble liver antigen
