# Supplementary material for: Antibodies against multiple post-translationally modified proteins aid in diagnosis of autoimmune hepatitis and associate with complete biochemical response to treatment
Source: Front Med (Lausanne). 2023 Jul 25;10:1195747. doi: 10.3389/fmed.2023.1195747 (PMC10411548; doi:10.3389/fmed.2023.1195747)
Supplement: Supplementary file 1 [file Presentation_1.pdf]

Supplementary files.

Antibodies against multiple post-translationally modified proteins aid in diagnosis of autoimmune hepatitis and associate with complete biochemical response to treatment

Michelle D. van den Beukel[1†], Anna E.C. Stoelinga[2†], Adriaan J. van der Meer[3], Stef van der Meulen[1], Lu Zhang[1], Maarten E. Tushuizen[2], Bart van Hoek[2] and Leendert A. Trouw[1*]
† These authors contributed equally to this work and share first authorship

***Correspondence**
Prof. L. A. Trouw, PhD
Department of Immunology, Leiden University Medical Center
Postbus 9600, 2300 RC Leiden
Telephone: 0031-715263869
Email: L.A.Trouw@lumc.nl

Supplementary Tables

Supplementary Table 1: Characteristics of AIH, PBC and PSC patients in the AILD cohort. Results are presented as n (%), or median (IQR). *AIH, autoimmune hepatitis; ALAT, alanine aminotransferase; ALP, alkaline phosphatase; AMA, anti-mitochondrial antibodies; ANA, anti-nuclear antibodies; ASAT, aspartate aminotransferase; SMA, smooth muscle antibody; CBR, complete biochemical response; GGT, gamma-glutamyl transferase; IgG, immunoglobulin gamma; LKM, Liver Kidney microsomal antibody; PBC, primary biliary cholangitis; PSC, primary sclerosing cholangitis; SLA, soluble liver antigen.* * pANCA (n=8), anti-parietal cell (n=1) ** based on cholangiographic findings with magnetic resonance/endoscopic retrograde cholangiopancreatography.

	**AIH (N=66)**	**PBC (N=10)**	**PSC (N=30)**
**Laboratory assessments**			
ALAT	338.0 (125.0 - 1057.5)	37.5 (28.0 – 76.8)	54.0 (37.0 – 114.0)
ASAT	420.0 (142.8 - 935.3)	50.5 (34.5 – 53.8)	48.0 (36.0 – 99.0)
IgG	24.8 (19.4-33.2)	-	-
ALP	178.5 (123.0 – 279.0)	277.0 (158.0 – 433.0)	229.0 (132.0 – 405.0)
GGT	212.0 (116.0 – 371.5)	147.0 (58.0 – 571.0)	166.0 (96.5 – 278.5)
**Positive antibodies**			Insufficient data
ANA	42 (63.6)	3 (33.3)	-
SMA	35 (53.0)	-	-
Anti-LKM	1 (1.5)	-	-
Anti-SLA	2 (3.0)	-	-
AMA	-	8 (80.0)	-
*Other**	9 (13.6)	-	-
**Large duct anomalies ****	N/A	N/A	29 (96.70)

Supplementary Table 2: Prevalence of antibodies against post-translationally modified proteins in patients with autoimmune (n=106) or non-autoimmune liver disease (n=101) and healthy controls (n=100). Results are presented as median [IQR] or n (%). *p<0.05, **<0.01 between HC and AILD #p<0.05, ##<0.01 between HC and non-AILD +p<0.05, ++<0.01 between AILD and non-AILD Autoimmune Liver Disease: AIH, PBC and PSC; non-Autoimmune Liver Disease: NAFLD, HCV, HBV, ALD, Combination, NASH. *AGE, advanced glycation end-product; AL, acetylated protein; aU/mL, arbitrary units per milliliter; CarP, carbamylated protein; Cit, citrullinated protein; IQR, interquartile range; MAA, malondialdehyde–acetaldehyde adduct; NT, nitrated protein;*

	**Healthy Controls n=100**				**Autoimmune Liver Disease n=106**				**Non-Autoimmune Liver Disease n=101**			
	**aU/mL [IQR]**		**n (% positive)**		**aU/mL [IQR]**		**n (% positive)**			**aU/mL [IQR]**	**n (% positive)**	
**Anti-MAA**	266,9	[200,4 – 370,2]	2	(2,0)	1036,0	[625,5 – 2119,6]**	72	(67,9)**	495,8	[315,2– 726,8]##, ++	27	(26,7)##, ++
**Anti-AGE**	88,9	[0,0 – 182,5]	4	(4,0)	234,5	[95,5 – 480,5]**	39	(36,8)**	130,0	[4,2 – 261,2]++	18	(17,8)##, ++
**Anti-CarP**	74,0	[1,5 – 157,9]	5	(5,0)	352,5	[213,5 – 633,0]**	50	(47,2)**	241,0	[83,5 – 422,0]##, +	28	(27,7)##, ++
**Anti-AL**	0,0	[0,0 – 9,8]	5	(5,0)	13,3	[2,8 – 30,7]**	20	(18,9)**	6,4	[0,0 – 10,0]++	10	(9,9)
**Anti-Cit**	1,3	[0,0 – 3,2]	6	(6,0)	3,1	[0,5 – 7,2]*	18	(17,0)*	1,6	[0,0 – 5,9]	15	(14,9)#
**Anti-NT**	108,0	[0,0 – 250]	6	(6,0)	269,0	[33,8 – 602,5]**	7	(6,6)	179	[0,0 – 501,5]	7	(6,9)

Supplementary Table 3: The association between the presence of anti-PTM antibodies and the three major autoimmune liver diseases. Results are presented as median (IQR) of n (%). Chi-2-tests were used to assess the difference between the presence of the specific manifestations and non-AILD patients. *p=0.006, **p<0.001. *AGE, advanced glycation end-product; AIH, autoimmune hepatitis; AL, acetylated protein; aU/mL, arbitrary units per milliliter; CarP, carbamylated protein; IQR, interquartile range; MAA, malondialdehyde–acetaldehyde adduct; non-AILD, non-autoimmune liver disease; PBC, Primary Biliary Cirrhosis; PSC, Primary Sclerosing Cholangitis*

	**AIH**		**PBC**		**PSC**		**Non-AILD (reference)**	
	**Yes, n=66**		**Yes, n=10**		**Yes, n=30**		**No, n=101**	
**Anti-MAA**								
aU/mL	1519.5	(760.0 – 2775.3)	787.6	(436.3 – 1669.7)	771.5	(545.9 – 1223.0)	495.8	(315.2– 726.8)
Positive	**51**	**(77.3)** **	5	(50.0)	**16**	**(53.3)** *	27	(26.7)
**Anti-AGE**								
aU/mL	349.0	(156.0 – 537.0)	76.5	(0.0 – 177.9)	172.5	(59.3 – 359.3)	130.0	(4.2 – 261.2)
Positive	**32**	**(48.5)** **	0	(0.0)	7	(23.3)	18	(17.8)
**Anti-CarP**								
aU/mL	475.5	(293.2 – 741.8)	148.7	(0.0 – 376.0)	230.5	(41.3 – 336.0)	241.0	(83.5 – 422.0)
Positive	**42**	**(63.6)** **	2	(20.0)	6	(20.0)	28	(27.7)
**Anti-AL**								
aU/mL	15.4	(4.7 – 33.4)	15.9	(0.8 – 24.8)	9.9	(0.2 – 28.0)	6.4	(0.0 – 10.0)
Positive	13	(19.7)	1	(10.0)	6	(20.0)	10	(9.9)

Supplementary Figure 1: Patients with AILD present with double or triple positivity for different anti-PTM antibodies compared to non-AILD and define a group of AIH patients harboring three anti-PTM antibodies. Upset plot indicating the frequency of combinations of anti-PTM antibodies in (A) non-AILD, (B) AILD, and (C) AIH. Horizontal bars represent the number of individuals in each of the anti-PTM antibodies of interest with a table on the right indicating single or combinations of anti-PTM antibodies. Vertical bars represent the number of patients positive for that anti-PTM antibody or combinations thereof.

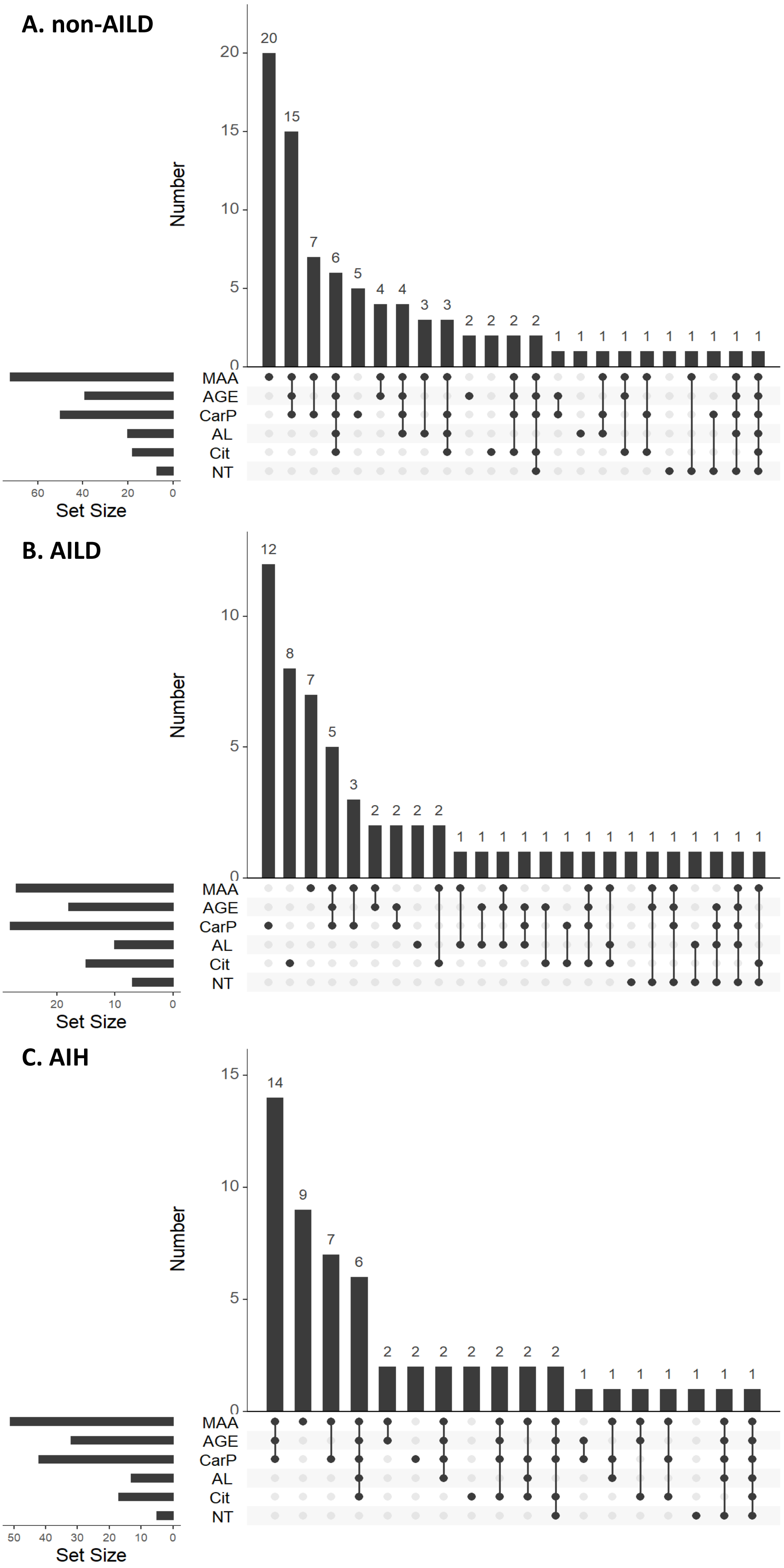

Supplementary Table 4: Detailed overview of correlation coefficients (including 95% confidence intervals) as provided in Figure 5. All confidence intervals were calculated using Fisher's transformation. *AGE, advanced glycation end-product; AI, autoimmune*; *AL, acetylated protein; ALAT, alanine aminotransferase; ALP, alkaline phosphatase; AMA, anti-mitochondrial antibodies; ANA, anti-nuclear antibodies; ASAT, aspartate aminotransferase; SMA, anti-smooth muscle antibody; CarP, carbamylated protein; CBR, complete biochemical response; CI, confidence interval; corr, correlation coefficient; IgG, immunoglobulin gamma; MAA, malondialdehyde–acetaldehyde adduct; LKM, Liver Kidney microsomal antibody; SLA, soluble liver antigen.*

	**Anti-MAA**		**Anti-AGE**		**Anti-CarP**		**Anti-AL**	
	corr	95% CI*	corr	95% CI*	corr	95% CI*	corr	95% CI*
**Demographics**								
Age	0.199	-0.06 – 0.43	0.230	-0.03 – 0.46	0.156	-0.11 – 0.40	-0.058	-0.31 – 0.20
Disease activity								
Cirrhosis	-0.36	-0.29 – 0.22	-0.183	-0.43 – 0.09	0.055	-0.21 – 0.31	-0.163	-0.42 – 0.12
Self-reported arthralgia	0.230	-0.03 -0.46	0.174	-0.10 – 0.42	0.006	-0.25 – 0.26	0.137	-0.15 – 0.40
Concurrent AI disease	0.110	-0.15 – 0.36	0.095	-0.18 – 0.35	0.110	-0.16 – 0.36	0.019	-0.26 – 0.30
**Transaminases**								
ALAT	0.024	-0.24 – 0.28	0.056	-0.21 – 0.31	0.207	-0.06 – 0.44	0.008	-0.25 – 0.27
ASAT	0.139	-0.13 – 0.38	0.135	-0.13 – 0.38	0.348	0.09 – 0.56	0.137	-0.13 – 0.38
**(Auto)antibodies**								
ANA	0.420	0.18 – 0.61	0.075	-0.19 – 0.33	0.272	0.01 – 0.49	0.143	-0.14 – 0.40
Anti-LKM1	-	-	-	-	-		-	
Anti-SLA	0.290	-0.03 – 0.51	-0.093	-0.39 – 0.23	0.229	-0.08 – 0.49	0.128	-0.21 – 0.43
SMA	-0.013	-0.31 – 0.29	0.015	-0.25 – 0.28	-0.154	-0.40 – 0.11	0.140	-0.14 – 0.40
IgG	0.529	0.29 – 0.70	0.248	-0.03 – 0.49	0.435	0.18 – 0.63	0.182	-0.10 – 0.43
**Follow-up**								
Time to CBR (years)	-0.269	-0.54 – 0.06	-0.337	-0.59 – 0.01	-0.266	-0.54 – 0.06	-0.191	-0.48 – 0.14
CBR 3 months	0.322	0.06 – 0.54	0.291	0.02 – 0.52	0.179	-0.09 – 0.42	0.260	-0.03 – 0.50
CBR 6 months	0.183	-0.08 – 0.42	0.085	-0.19 – 0.35	0.257	-0.01 – 0.49	0.303	0.02 – 0.54
CBR 12 months	0.328	0.07 – 0.54	0.386	0.12 – 0.59	0.341	0.08 – 0.55	0.270	-0.02 – 0.51
Liver transplantation	0.093	-0.17 – 0.34	0.190	-0.08 – 0.43	0.053	-0.21 – 0.31	0.081	-0.20 – 0.35
Mortality	0.028	-0.23 – 0.28	0.040	-0.23 – 0.30	0.165	-0.10 – 0.41	-0.042	-0.31– 0.24

Supplementary Table 5: Baseline characteristics of patients with AIH and at least 3 positive anti-PTM antibodies versus less than 3. Results are presented as n (%), mean ±SD or median (IQR). *AIH, autoimmune hepatitis; ALAT, alanine aminotransferase; ASAT, aspartate aminotransferase; CBR, complete biochemical response; IgG, immunoglobulin gamma.*

	**≥3 anti-PTM positive**	**<3 anti-PTM positive**	**p-value**
**Demographics**			
Patients	32 (55.2)	26 (44.8)	-
Female sex	24 (75.0)	19 (73.1)	0.87
Age diagnosis (years)	47.7 (±20.0)	44.8 (±18.9)	0.571
Simplified criteria for the diagnosis of AIH	8.0 (6.0 – 8.0)	7.0 (5.8 – 8.0)	0.19
Revised original criteria for AIH	17.9 (±2.6)	15.3 (±3.6)	<0.05*
Cirrhosis	12 (37.5)	11 (42.3)	0.71
Yes, compensated	8 (25.0)	6 (23.1)	-
Yes, decompensated	4 (12.5)	5 (19.2)	-
No cirrhosis	20 (62.5)	15 (57.7)	-
Self-reported arthralgia	7 (21.9)	5 (19.2)	0.81
Auto immune comorbidities	11 (34.4)	6 (23.1)	0.31
**Laboratory**			
ALAT	535.0 (186.0 – 1028.0)	333.0 (118.3 – 1136.0)	0.65
ASAT	580.0 (190.8 – 989.3)	304.0 (111.3 – 827.8)	0.29
IgG	28.8 (22.2 – 39.6)	21.0 (14.0 – 31.1)	0.02*
**Treatment started**			0.47
Azathioprine	1 (3.1)	0 (0.0)	-
Budesonide	0 (0.0)	2 (7.7)	-
Budesonide + azathioprine	2 (6.3)	1 (3.8)	-
Prednisolone	2 (6.3)	1 (3.8)	-
Prednisolone + azathioprine	25 (78.1)	20 (76.9)	-
Prednisolone + thioguanine	1 (3.1)	0 (0.0)	-
Thioguanine	0 (0.0)	1 (3.8)	-
Unknown	1 (3.1)	0 (0.0)	-
No treatment started	0 (0.0)	1 (3.8)	-
**Follow-up**			
Duration of follow-up	9.8 (4.9 – 18.2)	7.01 (4.0 – 12.26)	0.15
Time to CBR (years)	0.8 (0.3 – 2.3)	2.0 (0.8 – 4.1)	0.06
CBR 3 months	8 (25.0)	1 (3.8)	0.03*
CBR 6 months	9 (28.1)	3 (11.5)	0.12
CBR 12 months	13 (40.6)	3 (11.5)	0.01*
Liver transplantation	2 (6.3)	1 (3.8)	0.68
Mortality during follow-up	8 (25.8)	5 (19.2)	0.60
Switch in medication	15 (46.9)	10 (38.5)	0.36

Supplementary Table 6: Correlations between change in anti-PTM antibody levels and changes in aminotransferases and immunoglobulin gamma (IgG). All confidence intervals were calculated using Fisher's transformation. *AGE, advanced glycation end-product; AL, acetylated protein; ALAT, alanine aminotransferase; ASAT, aspartate aminotransferase; CarP, carbamylated protein; CI, confidence interval; corr, correlation coefficient; IgG, immunoglobulin gamma; MAA, malondialdehyde–acetaldehyde adduct.*

	**Δ Anti-MAA**			**Δ Anti-AGE**			**Δ Anti-CarP**			**Δ Anti-AL**		
	corr	p-value	95% CI*	corr	p-value	95% CI*	corr	p-value	95% CI*	corr	p-value	95% CI*
Δ ALAT	0.1	0.6	-0.3 – 0.5	0.28	0.2	-0.1 – 0.6	0.2	0.4	-0.2 -0.5	0.4	0.0*	0.0 – 0.78
Δ ASAT	0.2	0.4	-0.2 – 0.5	0.29	0.2	-0.1 – 0.6	0.3	0.1	-0.1 – 0.6	0.5	0.0*	0.18 – 0.7
Δ IgG	0.3	0.3	-0.2 – 0.7	0.6	0.007*	0.2 – 0.8	0.5	0.1	0.0 – 0.8	0.2	0.5	-0.3 – 0.6
